# Driver-passenger communicative stress and psychological distress among Chinese bus drivers: the mediating effect of job burnout

**DOI:** 10.1186/s12889-021-10618-x

**Published:** 2021-03-20

**Authors:** Zhihao Tu, Jingwen He, Na Zhou, Xinghua Shen

**Affiliations:** 1 Department of Nautical psychology, Faculty of Psychology, The Second Military Medical University, Shanghai, China; 2 Department of Medical Psychology, NO.96609 Hospital, Yinchuan, China; 3 Mental health education center, Facult of Psychology, The Second Military Medical University, Shanghai, China

**Keywords:** Stress, Job burnout, Mediation, Bus driver

## Abstract

**Background:**

This study aimed to investigate the relationship between driver-passenger communicative stress and psychological distress among bus drivers, as well as whether job burnout mediates the effect of driver-passenger communicative stress on psychological distress.

**Methods:**

A questionnaire consisting of a 12-item version of the General Health Questionnaire (GHQ-12), a one-item driver-passenger communicative stress scale, the Maslach Burnout Inventory-General Survey (MBI-GS), as well as sociodemographic and work factors, was distributed to 310 bus drivers in Shanghai, of which 307 completed it (99.0% response rate). A parallel multiple mediation model with bootstrap approach, was calculated to test the mediating effect.

**Results:**

Driver-passenger communicative stress, emotional exhaustion and cynicism were positively associated with psychological distress. Communicative stress was significantly positively linked with two of the three dimensions of burnout (emotional exhaustion and cynicism) and dependent variable. Emotional exhaustion and cynicism were positively associated with the dependent variable. The results indicate that emotional exhaustion and cynicism partially mediated the effect of communicative stress on psychological health, and that 60.0% of this effect can be explained by mediating effects, in which emotional exhaustion and cynicism weighed 63.2% and 36.8%, respectively.

**Conclusions:**

Communicative stress had effects on psychological distress among Chinese bus drivers, and job burnout was a mediator in this relationship.

## Background

On October 28, 2018, a bus carrying 15 people suddenly lost control and plunged into the Yangtze River, from a 20-m-high bridge in Wanzhou District, Chongqing City, China. All passengers lost their lives. According to the police investigation, a 48-year-old female passenger missed her stop and, as a consequence, first argued and then attacked the driver, causing the incident.

Previous studies on transportation safety and accident analysis, were mostly area-level or zone-level analysis, and focused mainly on macro predictors such as road network characteristics, land use patterns, and traffic information [1–4]. Fewer studies paid attention to more specific predictors, such as the ones referring to bus drivers and passengers [5].

The seriousness of the accident mentioned above, led to a greater concern on driver-passenger communication problems, and to an increased interest and care in bus drivers’ mental health.

Bus driving is a service-oriented occupation. Due to self-service buses becoming mainstream, bus drivers not only need to drive the vehicles, but also have to communicate with passengers, such as urging them to coin or swipe cards, maintaining order, answering passengers’ questions and so on. Inevitably, quarrels and even fights between drivers and passengers sometimes arise during communication, which sometimes result in complaints from the passengers and administrative punishments for the drivers. However, unlike in other industries, quarrelling or fighting between passengers and drivers, may threaten the safety of everyone involved or nearby. Thus, communicating with passengers can be a big occupational source of stress for bus drivers, and long-term exposure to this stressor may have an impact on their mental health. Previous studies found that work stress was positively related to the morbidity of fatigue, anxiety, depression and other mental health problems among bus drivers [6–8]. In addition, violence from passengers can cause bus drivers to develop acute stress disorder and post-traumatic stress disorder (PTSD) [9]. Furthermore, abundant studies have demonstrated that psychological distress in professional drivers was detrimental to their capacity to safely operate vehicles [10], and thus potentially increase the risk of road accidents [11]. However, few studies have focused on the driver-passenger communicative stress, and its relationship with psychological distress among bus drivers.

Job burnout is characterized by exhaustion, cynicism, and lack of achievement and productivity at work, and it mainly occurs among people-oriented professions [12]. Among these symptoms of burnout, exhaustion and cynicism constitute the core of job burnout [13]. Exhaustion means feelings of overextension and depletion of resources, while cynicism means negative or callous responses to job responsibilities [14]. Previous studies on burnout mostly focused on medical workers, especially nurses [15, 16]. However, as previously mentioned, drivers of self-service buses are required to deal extensively with passengers, which means they are susceptible to job burnout. Indeed, one study reported that the prevalence of severe and mild burnout was 3.6 and 30.1% respectively, among drivers and conductors in Mozambique [17]. Unfortunately, the prevalence of burnout among bus drivers in China is still not clear.

Abundant evidence showed that work stress was associated with burnout [18, 19]. According to the Job Demands-Resources (JD-R) Model, an unbalanced relationship between job demands and resources (i.e., demands outweigh resources), may cause job-related stress, which in turn can lead to job burnout and mental health issues [20]. Specifically, Hakanen et al. [21] found that the effect of job demands on health problems was mediated by job burnout (exhaustion and cynicism without professional inefficacy). Other studies also found that high demands and low resources may result in exhaustion and cynicism, but not affect professional efficacy [22, 23]. Many researchers argued that reduced professional efficacy may play a divergent role in the burnout process [13, 21, 24, 25].

Job burnout has been shown to have a wide and substantial impact on mental health, leading to an increase in alcohol consumption [26], sleep disturbance [27], and depression [28]. Yao et al. [29] found that work-related burnout was associated with the level of certain neurotransmitters in the cerebral cortex of medical workers, which may in turn cause psychological distress. Kasemy et al. [30] found that emotional exhaustion was associated with pro-inflammatory markers including IL6, TNFα, and CoQ10. Meanwhile, inflammation is a key factor in the developmental process of mental health problems [31], including depression [32], anxiety [33] and so on. Many studies suggested that the relationship of depression and burnout is bidirectional, which means they influence each other in the manner of a vicious cycle or downward spiral [34–36]. This bidirectional relationship may also occur between burnout and sleep disturbance [37]. Zhou et al. [38] suggested that Eysenck’s Psychoticism traits may moderate the relationships between burnout and anxiety mediated by coping styles. Interestingly, many studies showed that professional inefficacy dimension of burnout was also not related to health [13, 14]. Therefore, it is reasonable to speculate that job burnout (exhaustion and cynicism without professional inefficacy) may play a mediating role between communicative stress and psychological distress among bus drivers. To the best of our knowledge, no published studies have tested this hypothesis.

It is of great significance to investigate the current situation of bus drivers’ communicative stress as well as whether and how communicative stress influences drivers’ mental health. From point of bus companies and the government, based on these information and knowledge, they can formulate policies accordingly and take intervention measures to alleviate drivers’ communicative stress and improve their mental health. For the passengers, knowing these things will make them better understand the hardships of bus drivers and communicate with bus drivers more patiently in the future. All these mentioned above can prevent the Wanzhou tragedy from happenning again.

**Fig. 1:**
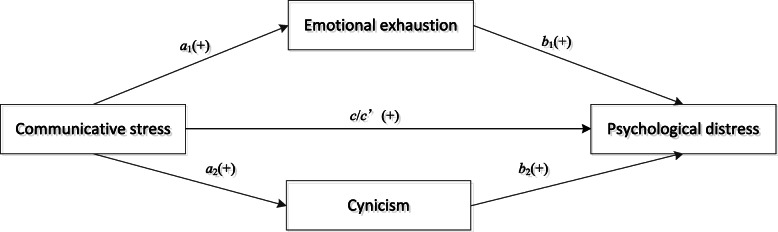
Hypothesized multiple mediator model of bus drivers’ communicative stress, job burnout, and psychological illness

Thus, our study aimed to investigate 1) whether driver-passenger communicative stress was associated with psychological distress among bus drivers; and 2) whether job burnout mediated the effects of driver-passenger communicative stress on psychological distress. We hypothesized that 1) driver-passenger communicative stress was positively associated with psychological distress among bus drivers; and 2) two dimensions of job burnout, exhaustion and cynicism, may mediate the effects of driver-passenger communicative stress on psychological distress. Figure 1 is the hypothesized multiple mediator model.

## Methods

### Study design and participants

Participants (*N* = 310) were bus drivers recruited from Shanghai BA-SHI Public Transportation and the data were collected between February and March 2019. In total, 307 questionnaires were fully completed and taken for analysis, yielding a 99.0% response rate. Bus drivers were informed of voluntary participation and anonymity. All participants signed written informed consent forms following a detailed explanation about the purpose of the study. The questionnaires were delivered and collected by researchers. This study received ethical approval from the Committee on Second Military Medical University. Study conducted is in accordance with Helsinki guidelines.

### Measures

#### Sociodemographic variables

The sociodemographic variables included age, educational level, marital status, and monthly family income (RMB). Educational level was categorized as ‘secondary school’, ‘high school’, and ‘higher education’. Marital status was categorized as ‘single’, ‘married’, and ‘divorced/widowed’.

### Measurement of job burnout

The Maslach Burnout Inventory – General Survey (MBI-GS) was used to measure participants’ burnout [39, 40]. This survey included three subscales (emotional exhaustion, cynicism, and professional efficacy). The subscales of emotional exhaustion (5 items) and cynicism (4 items) were used in this study. All the items were scored on a Likert scale from 0 (never) to 6 (every day). The example item for the subscales of emotional exhaustion and cynicism were “I feel emotionally drained from my work”, “I doubt the significance of my work” respectively. The Chinese version of the MBI-GS has been used widely in the Chinese population, and demonstrated satisfactory reliability and validity [41, 42]. In the present study, the Cronbach’s α for emotional exhaustion and cynicism were 0.94 and 0.91.

### Measurement of psychological distress

The 12-item version of the General Health Questionnaire (GHQ-12) is a commonly accepted measure of mental health and well-being [13]. The GHQ-12 asks participants to respond to 12 items describing their health “in general over the last month”. Each GHQ-12 item has 4-point responses: ‘not at all = 0’, ‘same as usual = 1’, ‘rather more than usual = 2’, and ‘much more than usual = 3’, such as “feeling nervous and strung up all the time” and “enjoying daily activities”.The GHQ-12 score, obtained by summing up the scores of the 12 items, measures the severity of minor psychiatric morbidity [43, 44]. The Chinese GHQ-12 is reliable and valid in the Chinese population [44, 45]. In the present study, Cronbach’s α was 0.86.

### Measurement of driver-passenger communicative stress

Until now, no measure of driver-passenger communicative stress had been used reliably. In this primary study, participants were asked to indicate their stress level on a ten-point scale, and one-item was used to measure driver-passenger communicative stress - “Please indicate the intensity of your driver-passenger communicative stress in recent month”, with response anchors low = 0, moderate = 5, and high = 10. This was not the first occasion where a one-item summary measure has been used. Indeed, another concept (subjective work stress) has been measured in an identical way in a previous study and it has been proven to be a valid tool [46]. Thus, it was acceptable to use this self-made scale in this study, to measure driver-passenger communicative stress.

### Statistical analysis

Statistical analysis was performed with SPSS (version 22.0). Descriptive statistics such as mean, range, standard deviation, and percentages were calculated. Differences in dimensions of burnout and psychological distress among different categorical demographic groups, were tested by one-way ANOVA or independent-sample t-test. Pearson correlation was used for testing the relationship between stress, burnout and psychological distress. A parallel multiple mediation model with bootstrap approach were calculated to test the mediating effect of emotional exhaustion and cynicism, in the relationship between communicative stress and psychological disorders. We applied the procedure recommended by Hayes [47] and the PROCESS macro of SPSS [48], to test the proposed parallel multiple mediation model. Four regression equations were set to estimate the effects of communicative stress on psychological distress:

1 $$ Y=i+ cX+e, $$

2 $$ {M}_1={i}_{M_1}+{a}_1X+{e}_{M_1}, $$

3 $$ {M}_2={i}_{M_2}+{a}_2X+{e}_{M_2}, $$

4 $$ \mathrm{Y}={i}_Y+{c}^{\prime }X+{b}_1{M}_1+{b}_2{M}_2+{e}_Y, $$

where *Y* = psychological distress, *X* = communicative stress, *M*_1_ = emotional exhaustion, *M*_2_ = cynicism, *c* = total effect of *X* on *Y*, *c*^′^ = direct effect of *X* on *Y*, *a*_*i*_= effect of *X* on *M*_*i*_ (*i* = 1 to 2), *b*_*i*_= effect of *M*_*i*_ on *Y* (*i* = 1 to 2), *i* represents intercept, and *e* represents error.

In the present study, the PROCESS macro estimated all coefficients with ordinary least squares (OLS) regression. The particular indirect effect of *X* on *Y* through *M*_*i*_ was represented by *a*_*i*_*b*_*i*_. The statistical significance of the specific indirect effects was tested by 95% bias-corrected accelerated confidence intervals (95% BCa CIs) on the basis of 5000 bootstrap samples. If the 95% BCa CIs does not contains 0, this particular indirect effect is considered statistically significant [49, 50].

## Results

Demographic and working characteristics of selected bus drivers and distributions of all variables in mediating model in categorical items are shown in Table 1.

**Table 1:** Demographic and working characteristics of participants, and the distributions of all variables in mediating model in categorical items

Variables	N (%)	CS		EE		Cynicism		GHQ-12	
		Mean (SD)	*p*	Mean (SD)	*p*	Mean (SD)	*p*	Mean (SD)	*p*
**Sex**									
Male	289 (94.1)	4.84 (3.27)	0.829	2.56 (1.58)	0.002	1.96 (1.46)	< 0.001	1.52 (2.44)	0.065
Female	18 (5.9)	4.67 (2.93)		1.39 (0.84)		0.72 (0.59)		0.44 (0.78)	
**Education**									
Secondary school	89 (29.0)	4.65 (3.22)	0.786	2.43 (0.17)	0.744	2.04 (0.15)	0.226	1.46 (0.25)	0.431
High school	172 (56.0)	4.86 (3.32)		2.55 (0.12)		1.89 (0.11)		1.56 (0.18)	
Undergraduate (above)	46 (15.0)	5.04 (3.01)		2.39 (0.23)		1.59 (0.21)		1.04 (0.35)	
**Marital Status**									
Married	260 (84.7)	4.85 (3.15)	0.653	2.51 (1.56)	0.509	1.93 (1.44)	0.170	1.47 (2.33)	0.858
Single	22 (7.2)	4.27 (3.64)		2.13 (1.50)		1.33 (1.22)		1.50 (2.94)	
Divorced/Widowed	25 (8.1)	5.12 (3.88)		2.61 (1.84)		1.97 (1.69)		1.20 (2.53)	

Note: *CS*  communicative stress, *EE*  emotional exhaustion, GHQ-12 = 12-item Chinese version of the General Health Questionnaire

Results of Pearson correlations among variables are displayed in Table 2. Communicative stress, emotional exhaustion, cynicism, and mental illness were positively associated with each other. Both age and tenure depicted positive relationships with emotional exhaustion and cynicism. Tenure was significantly correlated with communicative stress.

**Table 2:** Correlations, Means, and Standard Deviations Among Variables

Variable	M	SD	1	2	3	4	5	6	7
1. **CS**	4.83	3.24	1						
2. **EE**	2.49	1.57	0.67^***^	1					
3. **cynicism**	1.89	1.45	0.47^***^	0.79^***^	1				
4. **GHQ-12**	1.45	2.38	0.42^***^	0.51^***^	0.47^***^	1			
5. **Age**	45.60	8.53	0.10	0.15^**^	0.21^***^	0.11	1		
6. **Tenure**	20.92	11.60	0.15^*^	0.16^**^	0.18^**^	0.09	0.78^***^	1	
7. **Income**	8187.32	2438.76	−0.10	−0.06	−0.02	−0.07	0.14	0.00	1

Note: *N* = 307. CS = communicative stress, EE = emotional exhaustion, GHQ-12 = 12-item Chinese version of the General Health Questionnaire

^a *^p < .05. ^**^p < .01. ^***^p < .001

**Fig. 2:**
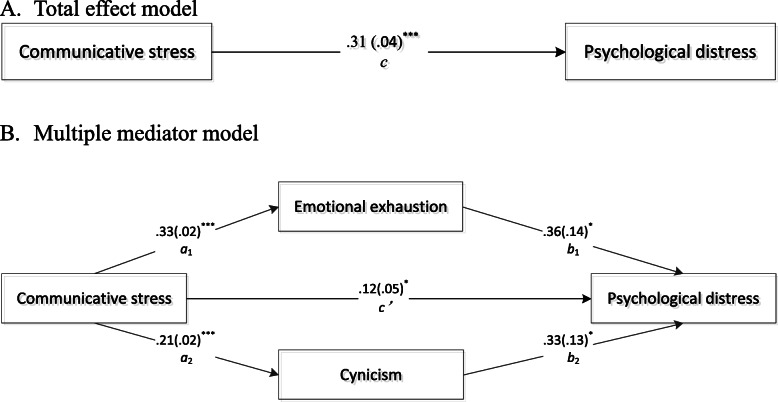
Multiple mediation with psychological illness as criterion. Path coefficients correspond to unstandardized parameter estimates (standard errors in parentheses). ^*^p < .05. ^**^p < .01. ^***^p < .001

The results of all the regression models set in the mediation analysis can be seen in Table 3. The path coefficients of multiple mediator model are shown in Fig. 2. Communicative stress was significantly positively linked with emotional exhaustion and cynicism and dependent variable. Emotional exhaustion and cynicism were positively associated with the dependent variable. The results indicate that emotional exhaustion and cynicism partially mediated the effect of communicative stress on psychological health, and that 61.3% of this effect can be explained by mediating effects, in which emotional exhaustion and cynicism weighed 63.2 and 36.8%, respectively. The total, direct and indirect effects are all presented in Table 4.

**Table 3:** Regression coefficients, standard errors, and model summary information for the presumed parallel multiple mediator model

Antecedent		*Y*			*M*_1_			*M*_2_			*Y*	
		*β*	*p*		*β*	*p*		*β*	*p*		*β*	*p*
***X***	*c*	0.307	< 0.001	*a*_1_	0.327	< 0.001	*a*_2_	0.209	< 0.001	*c*^′^	0.120	0.015
***M***_**1**_										*b*_1_	0.359	0.013
***M***_**2**_										*b*_2_	0.333	0.011
**Constant**	*i*	−0.029	0.897	${i}_{M_1}$	0.912	< 0.001	${i}_{M_2}$	0.879	< 0.001	*i*_*Y*_	−0.648	0.005
**Model Fit**		*R*^2^ = 0.174 *p* < 0.001			*R*^2^ = 0.454 *p* < 0.001			*R*^2^ = 0.218 *p* < 0.001			*R*^2^ = 0.282 *p* < 0.001

**Table 4:** Total, total indirect, specific indirect, and direct effects of the multiple mediator model

	Estimate	S.E.	[95% CI]
**Total effect**	0.31	0.04	[0.23, 0.38]
**Total indirect effect**	0.19	0.04	[0.11, 0.27]
**Direct effect**	0.12	0.05	[0.02, 0.22]
**Specific indirect effect**			
**Emotional exhaustion**	0.12	0.05	[0.02, 0.21]
**Cynicism**	0.07	0.03	[0.01, 0.14]

Note: *SE*  standard error, *CI*  confidence intervals

## Discussion

To the best of our knowledge, this was the first study examining the relationship between stress and mental health among bus drivers, as well as investigating the mediating effect of job burnout between the two.

### Direct effect of communicative stress on mental health

More precisely, we found that driver-passenger communicative stress was positively associated with psychological distress. This study also demonstrated that emotional exhaustion and cynicism partially mediated the effect of driver-passenger communicative stress on mental illness. These findings were as expected, and in line with previous studies. For example, Useche et al. [11] found that overall work-related stress was associated with mental health issues among professional drivers. Another study investigated the relationship between nine major types of work stressors (driver-passenger communicative stress was not included) and biomarkers of stress, 24-h urine cortisol and serum dehydroepiandrosterone-sulfate (DHEA-S), among bus drivers in Taiwan [51]. The results showed that only “poor relationship with supervisor” and “imbalanced salary and bonus” were significantly associated with biomarkers of stress. Although previous studies have neglected the effect of driver-passenger communicative stress on psychological distress, the results of our study showed that communicating with passengers is a big work-related stressor for bus drivers, and can indeed influence their mental health.

### Indirect effect of communicative stress on mental health

As mentioned above, our results also show that emotional exhaustion and cynicism partially mediate the effect of driver-passenger communicative stress on psychological health. Indeed, bus drivers who experience a higher level of communicative stress are more likely to show symptoms of exhaustion and cynicism, which in turn can affect their mental health. In this process, reduced professional efficacy did not seem to play an important role. The findings were as expected and agreed with previous studies. For example, Chen et al. [52] studied 1029 young nurses, and found that job burnout had a mediating effect in the relationship between job stress and mental health.

#### Effect of communicative stress on burnout

Maslach et al. [24] provided a sophisticated theoretical framework to understand job burnout in their influential review article. This framework identifies six kinds of mismatch contributing to job burnout: context, workload, control, reward, community, fairness, and values. In the present study, the path from communicative stress to job burnout may be attributed to the mismatch of fairness. In fact, bus drivers can be described as a vulnerable group, when they get involved in a dispute with passengers. As a matter of fact, even when it is the passenger who is unpleasant and impolite, as soon as the bus company receives the complaint from the passenger, the incident will be recorded and the driver will be warned and/or punished. Furthermore, on some occasions, drivers are forced to endure violence from passengers while driving, as to not risk a serious road accident. Thus, a feeling of unfairness may ensue within driver-passenger communication. Maslach et al. [24] suggested that a lack of fairness may aggravate burnout in at least two ways. First, both personal and colleagues’ experiences of unfair treatment can be emotionally exhausting. Second, unfairness may raise a deep sense of cynicism related to the job [24]. From the view of JD-R model, dealing with passengers while driving can carry a high emotional demand and overall workload. Unfortunately, there are not enough job resources for bus drivers on the road, to cope with the high job demands. This unbalanced relationship between job demands and resources can result in stress and burnout.

#### Effect of burnout on mental health

The effect pathway from burnout to psychological distress has been extensively studied, and many variables seem to play a mediating role in this., for example, resilience and emotional regulation [52, 53]. In addition, DNA methylation may also be involved in this process [18]. It is worth noting that many researchers have argued the occurrence of an extensive overlap between burnout and depression [54, 55]. Indeed, Orosz et al. [56] found that burnout cannot be distinguished from depression from biological indicators (brain-derived neurotrophic factor (BDNF), heart rate variability (HRV), and hippocampal volume). However, other researchers believe that the apparent overlap between burnout and depression is, instead, a result of different definitions of burnout [54]. A longitudinal study showed that burnout (exhaustion and cynicism) was a precursor of mental illness [13]. Thus, the issue of burnout-depression overlap needs more in-depth studies in the future [54].

### Limitations

Several limitations of the present study must also be addressed. First, this was a cross-sectional study, which cannot evaluate the temporality and causality. Even though we used mediation analysis, the causal relationships among job burnout, communicative stress, and psychological disorder, cannot be determined in the present study. Second, stress, job burnout and psychological distress were all measured by self-reported questionnaires, and the results may be affected by response bias. Third, despite the extensive level of adjustment in our study, the possibility remains that unmeasured confounders such as coping style, personality and physical health could explain part of the association between stress and psychological distress [57, 58]. Thus, the mediating effect of burnout (61.3% in this study) may be over rated. Lastly, fourth, we used only one item to measure driver-passenger communicative stress, which might not be very reliable. In addition, the item actually measures driver-passenger communicative stress in recent month. However, Metzenthin et al. [46] argued that one-item subjective work stress assessment tool was more accurate measuring contemporaneously than retrospectively.

### Implications for research and practice

Driver-passenger communication problem is a major threat to traffic safety, but it is always neglected by scholars and administration. To our best knowledge, this research was the first study focusing on the negative effect of driver-passenger communicative stress on mental health of bus drivers. From acedemic point of view, we suggest that, first, future researchers should develop a psychometrically sound measurement of driver-passenger communicative stress. Second, future researchers should design additional longitudinal studies to determine whether or not the causal relationships among job burnout, communicative stress, and mental illness exist. Third, this study showed that Chinese bus drivers are susceptible to burnout and suffer mental health problems. Thus, more studies should be conducted focunsing on bus drivers’ well-being. From practical point of view, the government and bus companies should take actions to protect bus drivers. The present study showed that driver-passenger communicative stress is harmful to drivers’ mental health and job burnout plays an important role in this process. Driver-passenger communicative stress mainly comes from the driver’s weak position relative to passengers. The favoritism of passengers by the government and bus companies makes matters worse and further leads to feeling of unfairness and burnout among drivers. Thus, the protection of bus drivers and penalties for irrational passengers should be strengthened both legislatively and administratively, which can relieve communicative stress at the source. In addition, this move can improve drivers’ sense of fairness and reduce job burnout, which can in turn enhance their mental health.

## Conclusions

The present study found that driver-passenger communicative stress had negative effects on job burnout and psychological disorders among Chinese bus drivers. Meanwhile, psychological disorders are associated with burnout. According to the result of mediation analysis, job burnout has a positive mediating effect on the path from driver-passenger communicative stress to psychological disorders. This result means that the more driver-passenger communicative stress bus drivers put up with, the higher level of burnout they suffer, which in turn leads to more symptoms of psychological disorders. The mediating effect of burnout can explain 60% of the total effect.

## Data Availability

All data generated or analysed during this study are available from the corresponding author on reasonable request.

## Abbreviations

GHQ-12: The General Health Questionnaire
MBI-GS: The Maslach Burnout Inventory-General Survey
PTSD: Post-traumatic stress disorder
JD-R: the Job Demands-Resources
OLS: Ordinary least squares
BC: Bias-corrected
CI: Confidence intervals
CS: Communicative stress
EE: Emotional exhaustion
PE: Professional efficacy
SE: Standard error
DHEA-S: Serum dehydroepiandrosterone-sulfate
BDNF: Brain-derived neurotrophic factor
HRV: Heart rate variability
